# A-synuclein prion strains differentially adapt after passage in mice

**DOI:** 10.1371/journal.ppat.1012746

**Published:** 2024-12-06

**Authors:** Sara A. M. Holec, Chase R. Khedmatgozar, Shelbe J. Schure, Tiffany Pham, Amanda L. Woerman

**Affiliations:** 1 Department of Microbiology, Immunology, and Pathology, Prion Research Center, Colorado State University, Fort Collins, Colorado, United States of America; 2 Department of Biology, University of Massachusetts Amherst, Amherst, Massachusetts, United States of America; National Institutes of Health, NIAID, UNITED STATES OF AMERICA

## Abstract

In patients with synucleinopathies, the protein α-synuclein misfolds into multiple conformations, each of which determines whether a patient develops multiple system atrophy (MSA) or one of three Lewy body diseases (LBDs). However, patients may also first present with pure autonomic failure, which strictly impacts autonomic nerves in the periphery, which can then phenoconvert into MSA or a LBD. When neuroinvasion happens, it remains unknown if strain properties are retained or if strain adaptation occurs, even though neuroinvasion of some prion protein (PrP) strains is known to result in the emergence of novel PrP strain variants. To investigate this question in synucleinopathies, we inoculated TgM83^+/-^ mice, which express human α-synuclein with the A53T mutation, with a mouse-passaged MSA patient sample either intracranially (i.c.) or into the sciatic nerve (sc.n.), and compared the biochemical and biological properties of α-synuclein prions in the brains of terminal mice. Importantly, while i.c. and sc.n. transmission studies generated pathogenic α-synuclein with similar properties, both the primary and secondary passaged MSA samples had different infectivity profiles in a panel of α-syn140-YFP cells than the starting MSA patient sample, indicating that MSA prions adapt during initial passage in TgM83^+/-^ mice. Similarly, using i.c. inoculation of A53T preformed fibrils to study strain selection, we found both biochemical and biological evidence that mouse passage exerts a selective pressure on α-synuclein prions in which a sub-population of starting conformations emerges in terminal animals. Together, these findings demonstrate that similar conformational selective pressures known to impact PrP prion replication also impact replication of α-synuclein prions.

## Author summary

The protein α-synuclein can misfold into multiple shapes that self-template (i.e., becomes a prion) to cause a group of neurodegenerative disorders known as synucleinopathies. The misfolded shape that α-synuclein adopts, or prion strain, determines the disorder a patient will develop. While the selective pressures that impact this same phenomenon in classical prion diseases, which are caused by misfolding of the prion protein, have been the focus of research for several decades, little is known about the effect of strain adaptation and/or selection on α-synuclein prion strains. The studies reported here demonstrate that both conformational selective pressures impact α-synuclein prion replication, which has important consequences for diagnostic and therapeutic development.

## Introduction

Synucleinopathies are a group of progressive neurodegenerative diseases, including multiple system atrophy (MSA) and the Lewy body diseases (LBDs), that are caused by the formation and spread of α-synuclein prions throughout the central nervous system. The variety of clinical symptoms and diseases that arise from the self-templated misfolding of a single protein can be explained by the strain hypothesis, which proposes that α-synuclein misfolds into multiple unique conformations, or strains, each of which gives rise to a distinct disease [1–8]. Research over the last decade has generated substantial biological, biochemical, and structural evidence supporting this hypothesis, with α-synuclein from MSA and LBD patient samples showing unique properties across multiple assays [1–3,7–14]. For example, MSA patient samples transmit neurological disease to TgM83^+/-^ mice, which express human α-synuclein with the A53T mutation, and replicate in α-syn140*A53T-YFP biosensor cells [10,15,16]. By comparison, LBD patient samples fail to propagate in cells unless first treated chemically or enzymatically [10,12,13,16] and have only shown a subclinical infection in TgM83^+/-^ mice in two studies [17,18].

While most research on patient-derived α-synuclein strains has focused on MSA and LBDs, a lesser known synucleinopathy, pure autonomic failure (PAF) or Bradbury-Eggleston syndrome, has remained understudied. PAF patients are defined by α-synuclein accumulation in peripheral autonomic nerves, which results in orthostatic hypotension, dizziness and lightheadedness, loss of bladder control, constipation, and abnormal sweating [19–21]. In a subset of PAF patients (ranging from 12–77%, depending on study cohort), what originates as a purely peripheral disorder phenoconverts into a central disease [20,22–25], with a roughly similar percentage of patients developing MSA or a LBD after neuroinvasion [25]. Notably, very little is known about how neuroinvasion of α-synuclein inclusions during phenoconversion impacts strain properties, though several studies in the prion field have shown that the site of disease origin, or exposure route, influences disease pathogenesis [26–28].

An increasing body of research on the prion protein (PrP) has shown that strain adaptation occurs in response to selective pressures, including altering exposure route. While some prion strains lose their ability to cause disease via peripheral routes of inoculation [26], comparison of intracerebral (i.c.) with peripheral inoculation for other PrP strains showed that unique strain variants emerge during neuroinvasion [27,28]. Our initial studies investigating the potential for MSA neuroinvasion found that peripheral inoculation of MSA patient samples intraperitoneally or intramuscularly (either hind leg or tongue) induced neurological disease in TgM83^+/-^ mice, though with an extended incubation period compared to i.c. inoculation [6]. However, while we detected the presence of α-synuclein prions in the brains of terminal mice using cell assay, biochemistry, and neuropathology, we did not evaluate the maintenance of strain properties following neuroinvasion from a variety of peripheral tissues, leaving questions about the potential for strain adaptation to occur during this process.

As noted above, LBDs do not readily nor reliably replicate in our model systems, which hinders investigating the Lewy strain. To circumvent this, many groups use preformed fibrils (PFFs), which are α-synuclein fibrils made from recombinant protein under a variety of aggregation conditions. While debate exists about whether PFFs are able to accurately serve as a proxy for the LBD strain [29], PFFs can serve as an important tool to investigate differences in α-synuclein strain properties both *in vitro* and *in vivo* [7–9]. Our previous studies using either wild-type or mutant PFFs inoculated into transgenic mouse models yielded evidence that during mouse passage, the change in replication environment selects for a sub-population of PFF conformations, resulting in the emergence of strains with altered biological properties compared to the starting inoculum [7,9]. However, a more thorough investigation of strain selection is needed, given the widespread use of PFFs in both basic science research and diagnostic and therapeutic development.

In the work reported here, we investigate the role of strain adaptation during neuroinvasion and selection of sub-populations on α-synuclein prion biology. In combining our *in vivo* studies with an expanded panel of α-syn140-YFP cell lines that have an enhanced resolution for discerning biological differences between α-synuclein prion strains [30], we find evidence that bolsters the hypothesis that MSA prions adapt during replication in TgM83^+/-^ mice [31], regardless of inoculation route, which we hypothesize occurs as a result of deformed templating [32]. Moreover, we show that i.c. inoculation of A53T PFFs results in strain selection, with passaged PFFs exhibiting a highly distinct infectivity profile in α-syn140-YFP cells compared to the starting inoculum. Together, these studies demonstrate that several factors known to influence adaptation and selection of PrP prion strains also contribute to α-synuclein prion strain biology.

## Results

### Neuroinvasion of α-synuclein prions occurs via retrograde spread through the spinal cord

**Fig 1:**
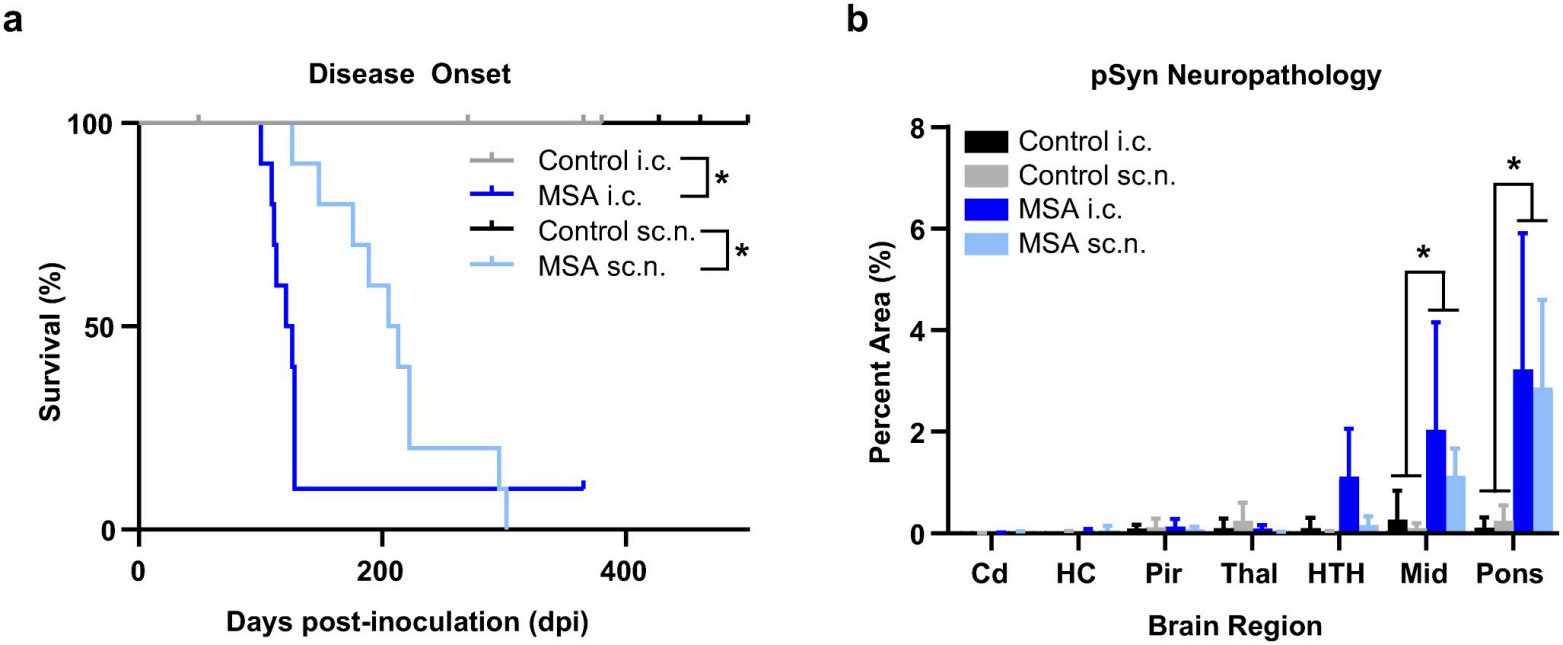
Mouse-passaged MSA induces neurological disease and α-synuclein neuropathology following central and peripheral inoculation. TgM83^+/-^ mice were inoculated with 20 μL of brain homogenate (standardized to 5 mg/mL total protein) of either mouse-passaged control (gray and black) or MSA patient sample (dark blue and light blue). Mice were injected either intracranially (i.c.) or into the sciatic nerve (sc.n.). (a) Kaplan-Meier plot showing disease onset in mice injected with control patient sample remained asymptomatic until 380 days post-inoculation (dpi; i.c.) or 500 dpi (sc.n.), while mice injected with passaged MSA developed neurological signs at 118 ± 10 dpi (i.c.) and 210 ± 56 dpi (sc.n.). (b) Quantification of phosphorylated α-synuclein pathology (EP1536Y primary antibody, 1:1,000 dilution) in the caudate (Cd), hippocampus (HC), piriform cortex and amygdala (Pir), thalamus (Thal), hypothalamus (HTH), midbrain (Mid), and pons following i.c. or sc.n. inoculation with control or MSA patient samples. Data are shown as mean ± SD. * = *P* < 0.05.

We previously showed that MSA prions transmit clinical disease to the TgM83^+/-^ mouse model via peripheral inoculation routes, including intraperitoneal and intramuscular inoculation [6]. However, while our studies confirmed the presence of α-synuclein prions in the brains of terminal animals using biological, biochemical, and neuropathological assessments, it was unclear if MSA prions retain strain properties or if they undergo adaptation when forced to enter the brain via a peripheral inoculation route. To determine if neuroinvasion from the periphery alters MSA strain properties, we compared sciatic nerve (sc.n.) injection with direct intracranial (i.c.) inoculation in TgM83^+/-^ mice. In these studies, we used both a control and an MSA patient sample (MSA16) that were previously passaged through TgM83^+/-^ mice to remove the confounding effect of genotype differences between agent and host from our analyses. Animals injected with the passaged control sample remained asymptomatic through 380 days post-inoculation (dpi; i.c.) and 500 days post-injection (sc.n.; Fig 1A). By comparison, the passaged MSA sample induced clinical disease in 118 ± 10 dpi following i.c. inoculation (*P* < 0.05) and 210 ± 56 dpi following sc.n. inoculation (*P* < 0.05). Consistent with our previous studies [6], the peripheral exposure increased incubation period by 78% compared to i.c. inoculation. Symptomatic mice exhibited typical clinical signs associated with the MSA strain, including bradykinesia, loss of forepaw strength, bilateral hindlimb clasping progressing to paralysis, and kyphosis, regardless of inoculation route.

**Fig 2:**
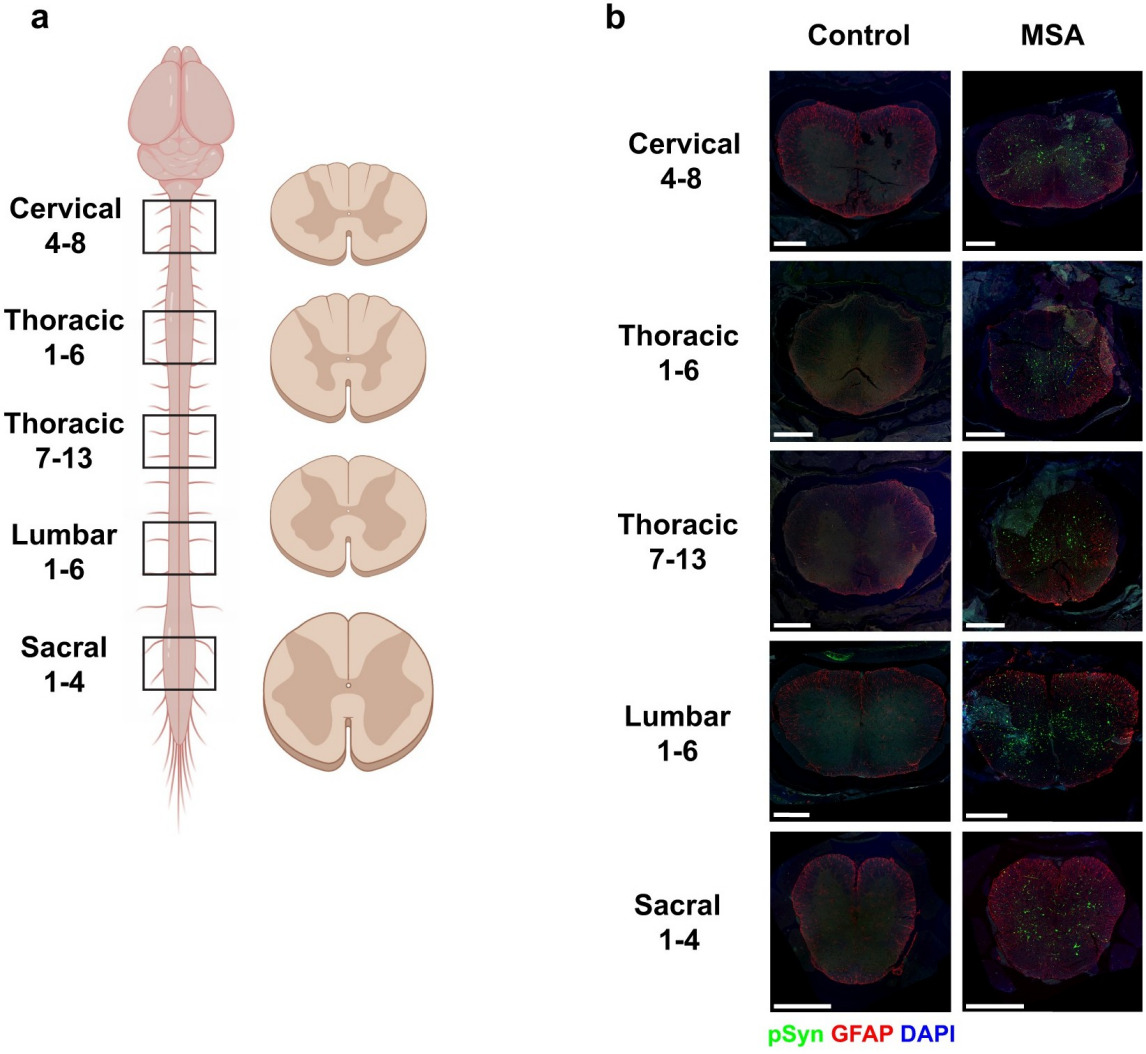
Mouse-passaged MSA prions induce robust bilateral α-synuclein pathology in the spinal cord of TgM83^+/-^ mice after sciatic nerve inoculation. TgM83^**+/-**^ mice were inoculated with 20 μL of brain homogenate (standardized to 5 mg/mL total protein) of either mouse-passaged control or MSA patient sample into the sciatic nerve (sc.n.). Spinal cords were collected from control-inoculated mice 500 days post-inoculation (dpi) and from MSA-inoculated mice following onset of clinical disease. Samples were analyzed for the presence of α-synuclein pathology (EP1536Y primary antibody; 1:1,000) throughout the length of the spinal cord. (a) Schematic showing the spinal cord regions analyzed. (b) Representative images from the cervical, thoracic (levels 1–6 and 7–13), lumbar, and sacral regions of the spinal cord from one control- and one MSA-inoculated mouse. Phosphorylated α-synuclein in green, glial fibrillary acidic protein (GFAP; 1:500) in red, and DAPI in blue. Scale bar, 400 μm. Fig 2a was made with Biorender.com.

Following the onset of these progressive clinical signs, brains were collected and bisected down the midline with one half fixed in formalin for histology and the other frozen for biochemistry. Neuropathology was assessed in symptomatic animals using the EP1536Y antibody, which detects α-synuclein phosphorylated at Ser129. Both i.c. and sc.n.-injected mice developed pathology throughout the hindbrain, including the pons, midbrain (Mid), and hypothalamus (HTH; Fig 1B and S1 Fig). We also assessed spinal cords from sc.n.-injected mice for astrogliosis and the deposition of phosphorylated α-synuclein. Sections of the spinal cord were collected from the sacral, lumbar, thoracic, and cervical regions (Fig 2A), with representative images shown in Fig 2B. The spinal cords of control-injected mice remained free of α-synuclein inclusions whereas MSA-injected mice developed extensive pathology throughout the spinal cord, which was accompanied by an increase in astrogliosis. Interestingly, spinal cord pathology was bilaterally symmetrical and located throughout multiple neuronal tracts, suggesting that spread to contralateral tracts occurs early during neuroinvasion (Fig 2B).

**Fig 3:**
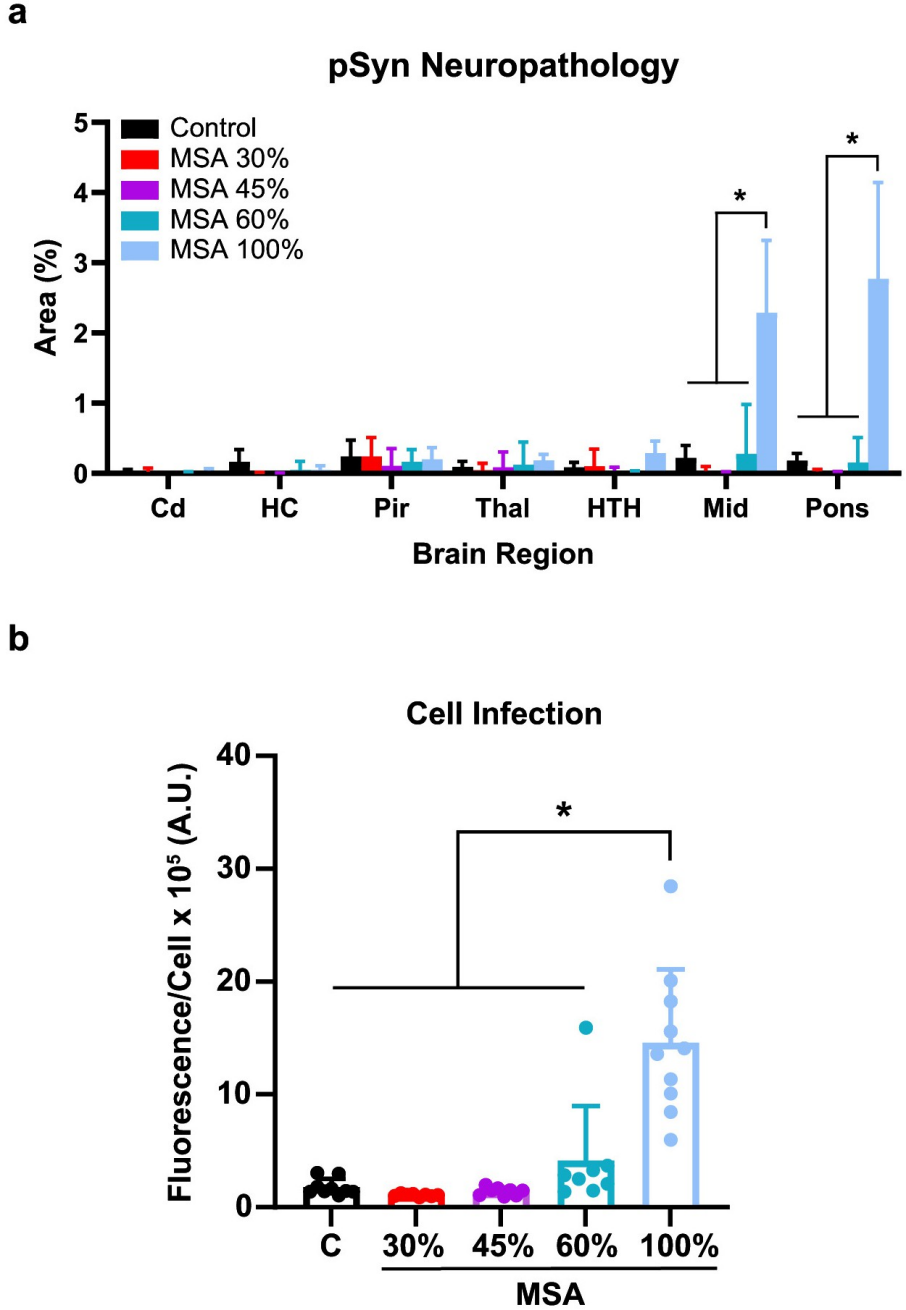
Neuroinvasion of MSA α-synuclein prions consistently occurs after 60 percent of the incubation period. TgM83^**+/-**^ mice were inoculated with 20 μL of brain homogenate (standardized to 5 mg/mL total protein) of either mouse-passaged control (black) or MSA patient sample into the sciatic nerve (sc.n.). After inoculation, mice were sacrificed at 30% (63 dpi, red), 45% (95 dpi, purple), or 60% (126 dpi, teal) of the MSA incubation period, or at onset of terminal disease (light blue). Brains were collected and analyzed for the presence of α-synuclein prions using (a) immunohistochemistry (EP1536Y primary antibody; 1:1,000) and (b) the α-syn140*A53T–YFP cell assay. (a) Quantification of pathology in the caudate (Cd), hippocampus (HC), piriform cortex and amygdala (Pir), thalamus (Thal), hypothalamus (HTH), midbrain (Mid), and pons. (b) Quantification of α-synuclein prion infectivity in α-syn140*A53T–YFP cells (× 10^5^ arbitrary units [A.U.]). Data are shown as mean ± SD. * = *P* < 0.05.

To assess disease progression along the spinal cord, we chose three intermediate timepoints based on the 100% incubation period for MSA (210 ± 56 dpi; Fig 1A). These timepoints– 30% (63 dpi), 45% (96 dpi), and 60% (126 dpi)–were selected based on previous studies investigating PrP^Sc^ neuroinvasion in hamsters [33]. Eight TgM83^+/-^ mice inoculated with passaged MSA were collected at each timepoint. The spinal cord and fixed half-brain from each mouse were assessed for α-synuclein pathology while the frozen half-brain was homogenized for biological and biochemical assays. No pathology was detected in the spinal cords from mice injected with the passaged control sample, nor from the MSA-inoculated mice collected at the 30% and 45% timepoints (Table 1). Two mice from the 60% MSA cohort were positive for α-synuclein pathology throughout the spinal cord, as were all mice from the terminal MSA group. Similarly, no significant pathology was detected in the brains from control-inoculated mice nor from any of the intermediate MSA timepoints (Fig 3A), though pathology was present in the terminal cohort, as reported above. By comparison, when homogenates from the frozen half-brains were analyzed using our α-syn140*A53T-YFP prion quantification assay, α-synuclein prions were detected in one of the 60% timepoint mice and all terminal animals (Fig 3B and S1 Table). These data coincide with our previous findings using a larger number of samples and mice to show that MSA prion formation precedes the detection of neuropathological inclusions in the brain [34]. Notably, despite the extension in incubation period, these findings also demonstrate that peripheral inoculation of MSA prions yields similar clinical signs and neuropathology as i.c. inoculation in the TgM83^+/-^ mouse model, suggesting that MSA prions retain strain properties upon neuroinvasion.

**Table 1:** Phosphorylated α-synuclein pathology in the brain and spinal cord of TgM83^+/-^ mice inoculated with passaged MSA.

Animals positive for pSyn pathology (n/n_0_*)						
Level	Segment	Control	MSA 30%^a^	MSA 45%^b^	MSA 60%^c^	MSA 100%
Brain	na	0/8	0/8	0/8	2/8	7/7
Cervical	4–8	0/8	0/8	0/8	2/8	7/7
Thoracic	1–6	0/8	0/8	0/8	2/8	7/7
Thoracic	7–13	0/8	0/8	0/8	2/8	7/7
Lumbar	1–6	0/8	0/8	0/8	2/8	7/7
Sacral	1–4	0/8	0/8	0/8	2/8	7/7

*n/n_0_ animals positive for pSyn pathology over total animals in experimental group.

^a^The 30% group was collected 63 dpi.

^b^The 45% group was collected 96 dpi.

^c^The 60% group was collected 126 dpi.

### MSA strain properties are maintained following neuroinvasion from the periphery

**Fig 4:**
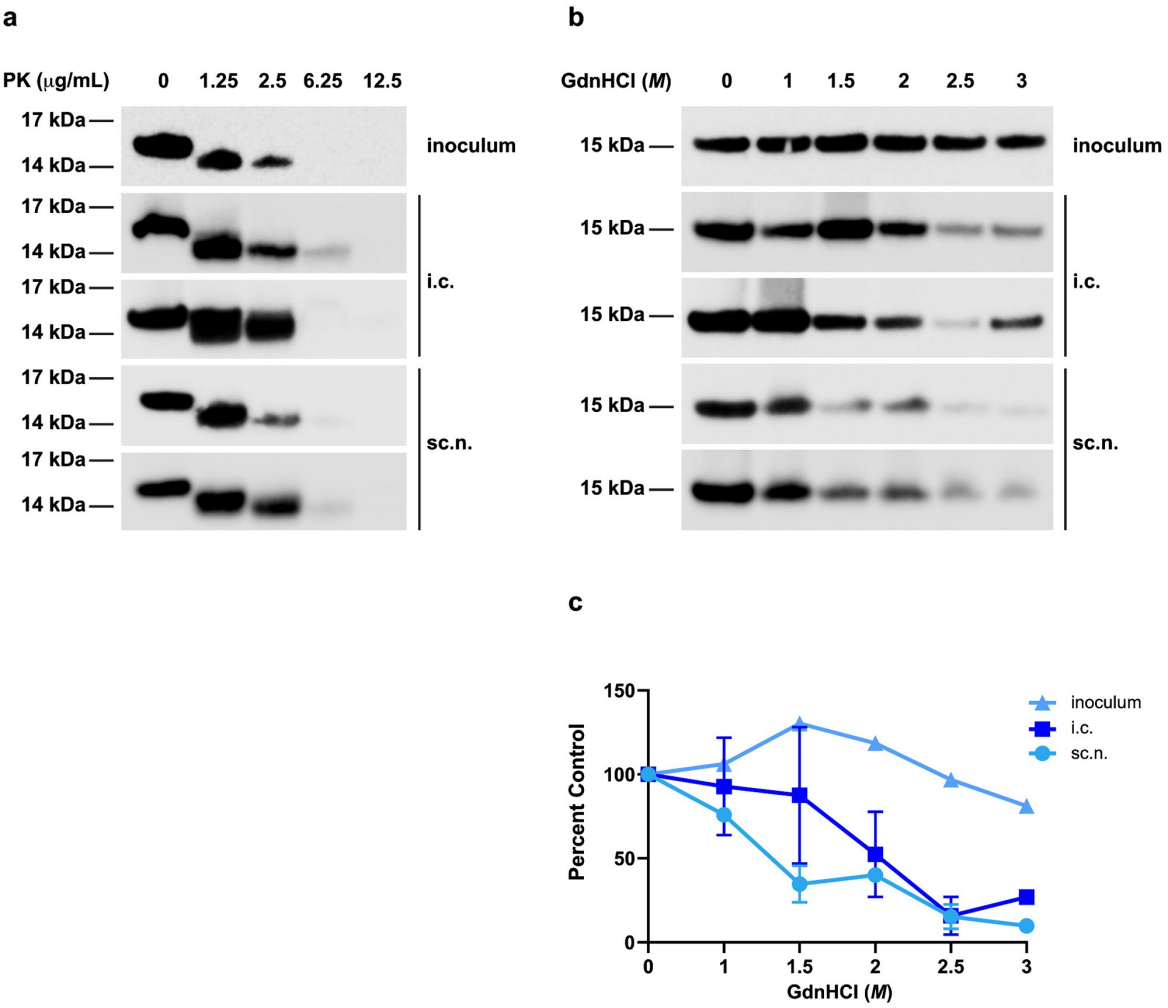
Α-synuclein prions retain biochemical stability regardless of inoculation route. TgM83^**+/-**^ mice were inoculated with 20 μL of brain homogenate (standardized to 5 mg/mL total protein) of mouse-passaged MSA patient sample intracranially (i.c.) or into the sciatic nerve (sc.n.). Frozen half-brains were collected from terminal mice and homogenized in 1× DPBS. The mouse-passaged MSA inoculum or homogenates from terminal i.c. or sc.n. injected mice were adjusted to 1 mg of total protein before biochemical analyses. (a) Western blot of remaining phosphorylated α-synuclein from mouse-passaged MSA inoculum (top), i.c. injected mice (middle two immunoblots from two different mice), and sc.n. injected mice (bottom two immunoblots from two different mice) after 30 min digestion with 0, 1.25, 2.5, 6.25, or 12.5 μg/mL of Proteinase K (PK). (b) Western blot of remaining phosphorylated α-synuclein from mouse-passaged MSA inoculum (top), i.c. injected mice (middle two immunoblots from two different mice), and sc.n. injected mice (bottom two immunoblots from two different mice) after 2 h incubation with 0, 1, 1.5, 2, 2.5, or 3 *M* guanidine hydrochloride (GdnHCl). All blots were probed with EP1536Y primary antibody (1:4,000). (c) Quantification of protein abundance after GdnHCl denaturation relative to incubation with 0 *M* GdnHCl from the blots shown in (b). Data are shown as mean ± SD.

For some PrP^Sc^ strains, such as chronic wasting disease, it is well documented that strain biology is altered or impacted by exposure route [26–28,35]. To thoroughly investigate strain property maintenance in i.c.- versus sc.n.-inoculated animals, we used a combination of traditional prion strain typing methods assessing biochemical stability in the presence of proteinase K (PK) or guanidine hydrochloride (GdnHCl), as well as our panel of α-syn140-YFP cell lines that quantify the biological activity of α-synuclein prion strains. In our biochemical analyses, frozen half-brains from the terminal cohorts, as well as the starting inoculum, were digested with PK or denatured using GdnHCl prior to immunoblotting. Regardless of inoculation route, MSA prions were completely resistant to digestion with 1.25 μg/mL PK, partially resistant to 2.5 μg/mL PK, and largely degraded by 6.25 μg/mL PK (Fig 4A). The MSA inoculum was also resistant to denaturation by 3 *M* GdnHCl; this trend was largely preserved after passage, regardless of inoculation route, though we observed a decrease in the amount of resistant phosphorylated α-synuclein compared to the inoculum (Fig 4B and 4C).

**Fig 5:**
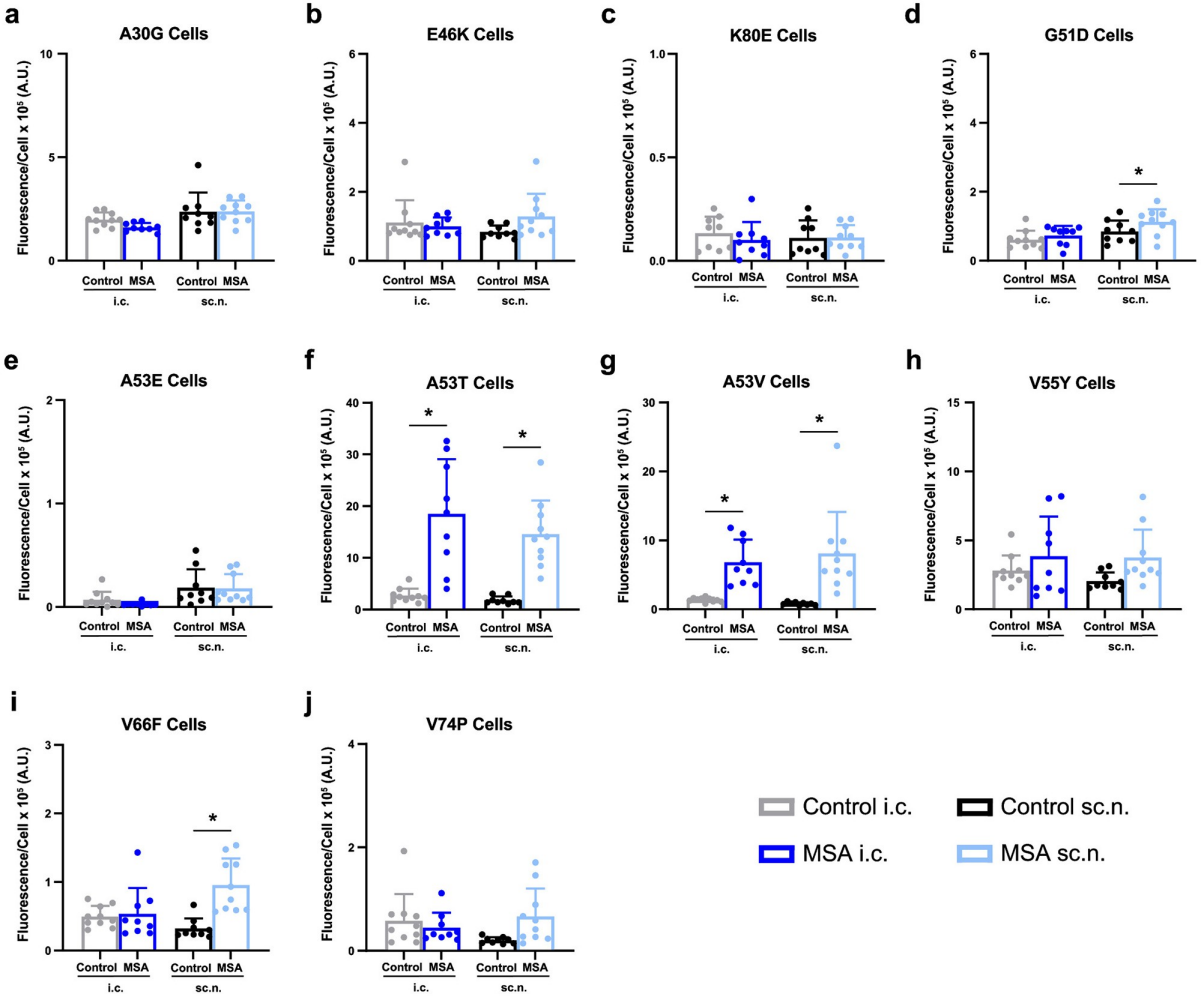
Α-synuclein prions isolated from i.c.- and sc.n.-injected mice have a similar infectivity profile in a panel of α-syn140-YFP cells. TgM83^+/-^ mice were inoculated with 20 μL of brain homogenate (standardized to 5 mg/mL total protein) from either mouse-passaged control (gray and black) or an MSA patient sample (dark blue and light blue). Mice were injected either intracranially (i.c., left side of each graph) or into the sciatic nerve (sc.n., right side of each graph). Frozen half-brains were collected from terminal mice and homogenized in 1× DPBS. Phosphotungstic acid precipitation was used to isolate α-synuclein prions from homogenates, and the resulting pellets were incubated with HEK293T cells expressing α-syn140-YFP fusion proteins harboring one of the following mutations: (a) A30G, (b) E46K, (c) K80E, (d) G51D, (e) A53E, (f) A53T, (g) A53V, (h) V55Y, (i) V66F, or (j) V74P. Quantification of α-synuclein prion infectivity (× 10^5^ arbitrary units [A.U.]) shown as mean ± SD. * = *P* < 0.05.

We then used our panel of α-syn140-YFP cell lines to determine if neuroinvasion altered the ability of MSA prions to replicate using α-synuclein expressing a variety of mutations. We recently expanded this panel of cell lines to incorporate several additional mutations, which has enhanced our ability to resolve differences between α-synuclein prion strains [30]. We first tested the MSA patient sample used to generate the mouse-passaged MSA inoculum used here (MSA16), as well as a control patient sample (C2), alongside the two passaged inocula for their ability to replicate in each cell line (S2 Fig and S2 Table). MSA16 significantly infected cell lines expressing the A30G, G51D, A53T, A53V, V55Y, V66F, and V74P mutations (*P* < 0.05) while patient sample C2 did not infect in any of the cell lines tested. Similarly, the control inoculum was unable to replicate in any cell line whereas the mouse-passaged MSA sample significantly infected cell lines expressing the A53T and A53V mutations (*P* < 0.05) and produced insignificant, weak infection in the G51D cell line (*P* = 0.05; S2 Fig). Additionally, the A53E cell line showed a statistically significant infection (*P* < 0.05; S2 Fig), though the biological relevance of a change from 0.01 ± 0.01 x 10^5^ A.U. to 0.36 ± 0.28 x 10^5^ A.U. is below the typical threshold we use to define a biologically meaningful change in cell infection (defined by a measured 2 x 10^5^ A.U. increase in fluorescence/cell relative to the negative control sample). We then tested brain homogenates from the terminal i.c.- and sc.n.-injected mice in the same 10 cell lines (Fig 5 and S3 Fig and S3 Table). Consistent with the starting inoculum, samples from both groups of control-injected mice did not infect any of the α-syn140-YFP cells whereas samples from the MSA-injected mice infected some but not all of the cell lines tested. The PD-associated A30G mutation has been shown to accelerate α-synuclein fibrillization *in vitro* [36], however, this mutation blocked infection of our mouse-passaged MSA samples, regardless of injection site (*P* = 0.90, i.c.; *P* = 0.24, sc.n.; Fig 5A). We previously showed that the PD-associated E46K mutation [37] also blocks MSA propagation both *in vitro* and *in vivo* [7,8] by preventing the formation of a critical salt bridge between residues E46 and K80 [30]. Unsurprisingly, here we found that samples from the MSA-inoculated mice were unable to replicate in either the α-syn140*E46K-YFP cell line (*P* = 0.91, i.c.; *P* = 0.20, sc.n.; Fig 5B) or the α-syn140*K80E-YFP cell line (*P* = 0.80, i.c.; *P* = 1.0, sc.n.; Fig 5B). The G51D mutation [38] supported insignificant sparse aggregate formation from the MSA-inoculated i.c. mice (*P* = 0.56), whereas a similar low infection from the sc.n.-inoculated animals was significantly different from control (*P* < 0.05; Fig 5D). Notably, the two MSA-inoculated cohorts were not statistically different from one another (*P* = 0.18), consistent with the minimal change compared to control. Multiple mutations at residue 53 are known to cause PD (A53E, T, and V) [39–41]. Here, the A53E mutation blocked MSA prion replication (*P* = 0.54, i.c.; *P* = 0.18, sc.n.; Fig 5E) whereas samples from MSA-inoculated mice caused significant infection in the α-syn140*A53T-YFP and α-syn140*A53V-YFP cells, regardless of inoculation route (*P* < 0.05; Fig 5F and 5G). Cells expressing the V55Y mutation blocked significant infection by samples from MSA-inoculated mice, regardless of injection site (*P* = 0.49, i.c.; *P* = 0.24, sc.n.; Fig 5H). Similarly, the i.c.-inoculated MSA samples were unable to replicate in the α-syn140*V66F-YFP cell line (*P* > 0.99). The sc.n.-injected samples induced a significant, but not biologically meaningful, infection (*P* < 0.05; Fig 5I), which is supported by the lack of statistical difference between the two cohorts (*P* = 0.07). Finally, the V74P mutation also blocked replication of the MSA samples, regardless of inoculation route (*P* = 0.85, i.c.; *P* = 0.10, sc.n.; Fig 5J). Together, the data from traditional strain typing methods suggest that MSA α-synuclein prions maintain their stability regardless of inoculation route, which is supported by a cell infectivity profile that is consistent for the passaged MSA strain before and after i.c. and sc.n. inoculation. However, we observed a notable reduction in the number of α-syn140-YFP cell lines capable of supporting MSA prion replication after passage in the TgM83^+/-^ mice (Table 2).

**Table 2:** The infectivity of α-synuclein prions in α-syn140-YFP cell lines demonstrates MSA strain adaptation and conformational selection of A53T PFFs.

Mutation	Control	MSA16	MSA inoculum	Passaged MSA		PFF* inoculum	Passaged PFFs
				i.c	sc.n.		
A30G							
E46K							
K80E							
G51D							
A53E							
A53T							
A53V							
V55Y							
V66F							
V74P							

*PFFs contain the A53T mutation.

Red, negative infection; Blue, positive infection.

### TgM83^+/-^ mice select for a subset of A53T PFF strains following i.c. inoculation

**Fig 6:**
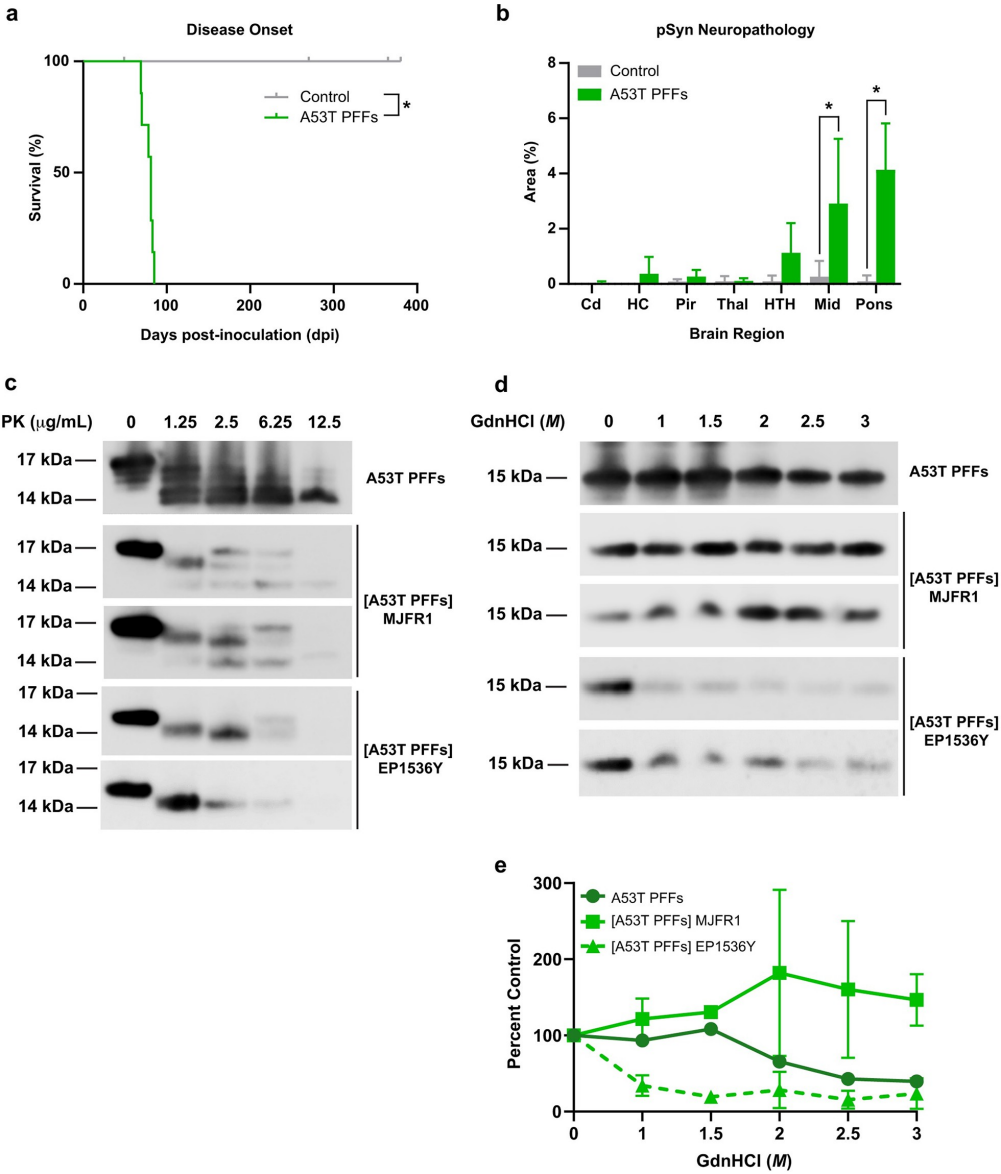
Mouse-passaged A53T PFFs show biochemical evidence of strain selection. TgM83^+/-^ mice were intracranially (i.c.) inoculated with 20 μL of brain homogenate (standardized to 5 mg/mL total protein) of mouse-passaged control patient sample (gray) or 1 mg/mL human α-synuclein PFFs with the PD-associated A53T mutation (green). (a) Kaplan-Meier plot showing disease onset in mice injected with control patient sample remained asymptomatic until 380 days post-inoculation (dpi; i.c.), while mice injected with A53T PFFs developed neurological signs at 78 ± 6 dpi. (b) Fixed half-brains were analyzed for phosphorylated α-synuclein pathology (EP1536Y primary antibody, 1:1,000 dilution) in the caudate (Cd), hippocampus (HC), piriform cortex and amygdala (Pir), thalamus (Thal), hypothalamus (HTH), midbrain (Mid), and pons. (c & d) Frozen half-brains were homogenized in 1× DPBS and adjusted to 1 mg of total protein before biochemical analyses, while A53T PFFs were diluted to 200 μg/mL. (c) Western blots of total α-synuclein from A53T PFF inoculum (top) and mouse- passaged A53T PFFs [A53T PFFs] probed for total (middle two blots) and phosphorylated α-synuclein (bottom two blots) from two different mice after 30 min digestion with 0, 1.25, 2.5, 6.25, or 12.5 μg/mL of Proteinase K (PK). (d) Western blots of total α-synuclein from A53T PFF inoculum (top) and [A53T PFFs] probed for total (middle two blots) and phosphorylated α-synuclein (bottom two blots) from two different mice after 2 hr incubation with 0, 1, 1.5, 2, 2.5, or 3 *M* guanidine hydrochloride (GdnHCl). (e) Quantification of protein abundance after GdnHCl denaturation relative to incubation with 0 *M* GdnHCl from the blots shown in (d). Quantification of the inoculum shown in olive green. (c & d) Membranes were probed using the MJFR1 primary antibody (1:12,500) or EP1526Y primary antibody (1:4,000). Data are shown as mean ± SD. * = *P* < 0.05.

Preformed fibrils (PFFs) are frequently used to study α-synuclein misfolding, strain biology, and disease pathogenesis, as well as for developing diagnostics and therapeutics for PD patients because they efficiently induce protein misfolding and clinical disease in model systems. However, a growing body of work highlights the variety of structures that α-synuclein PFFs can adopt, which can impact biological outcomes both *in vivo* and *in vitro* [42]. Despite these limitations, PFFs are often treated as a singular strain, which would require that the majority of misfolded α-synuclein exist in one conformation. Instead, recent findings from our lab suggest that transmission of mutant and wild-type PFFs to humanized mouse models of synucleinopathy selects for a subset of the initial fibril population [7,9]. To more carefully investigate these previous results, we interrogated PFF strain properties before and after passage in TgM83^+/-^ mice. Compared to control-inoculated mice, which remained asymptomatic through 380 dpi, i.c. inoculation of A53T PFFs induced the onset of clinical disease in 78 ± 6 dpi (*P* < 0.05), which is, on average, 40 days earlier than the clinical disease caused by i.c. inoculation of passaged MSA (Fig 6A). Following the onset of neurological signs, we assessed fixed half-brains from terminal animals and found that the A53T PFFs induced robust hindbrain deposition of phosphorylated α-synuclein accompanied by astrogliosis, which was absent in control-inoculated mice (Fig 6B and S4 Fig).

**Fig 7:**
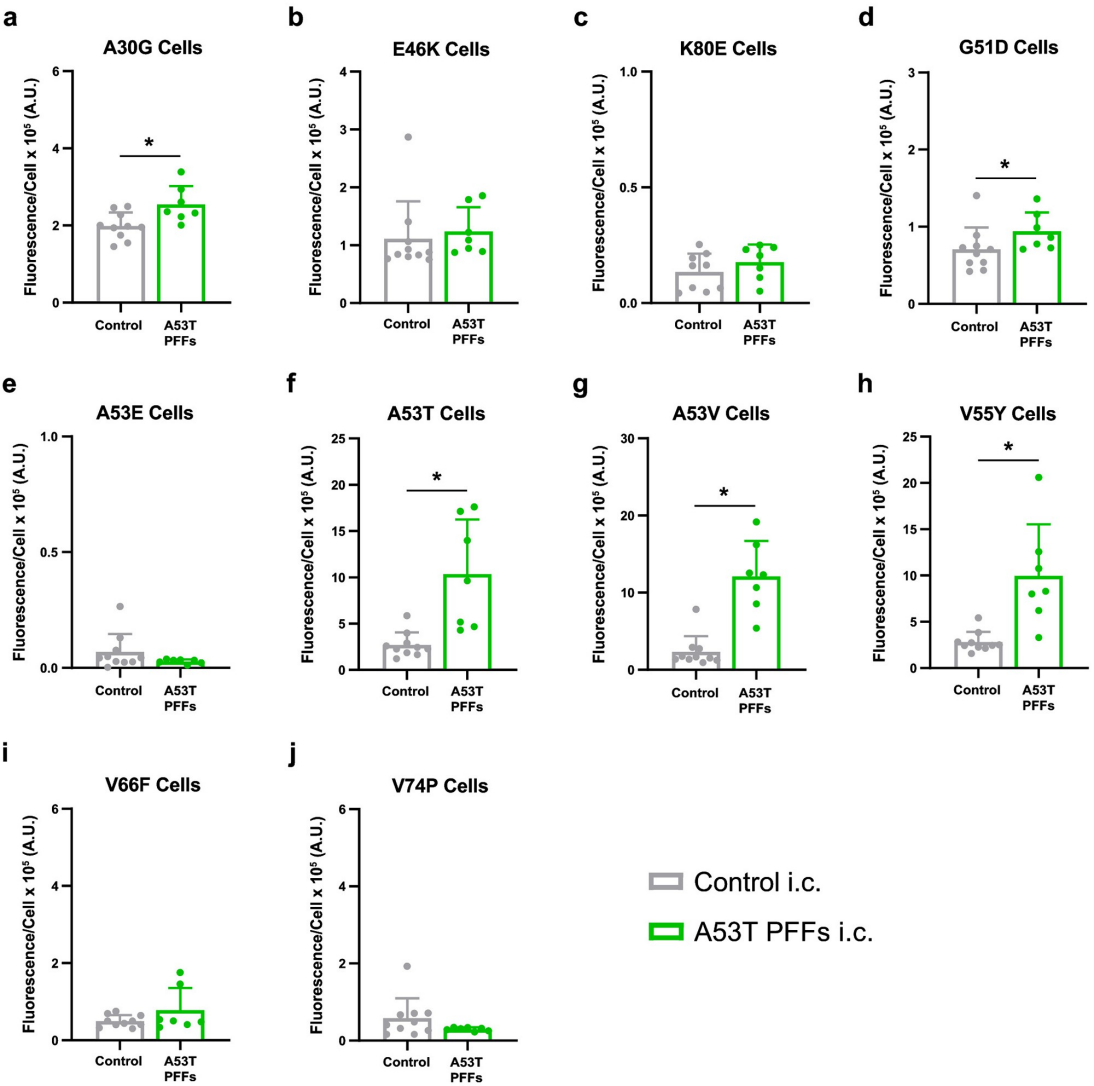
Mouse-passaged A53T PFFs have an altered infectivity profile in α-syn140-YFP cells. TgM83^+/-^ mice were intracranially (i.c.) inoculated with 20 μL of brain homogenate (standardized to 5 mg/mL total protein) of mouse-passaged control patient sample (gray) or 1 mg/mL human α-synuclein preformed fibrils (PFFs) with the PD-associated A53T mutation (green). Frozen half-brains collected from terminal mice were homogenized in 1× DPBS. Phosphotungstic acid was used to precipitate α-synuclein prions from homogenates, and the resulting pellets were incubated with HEK293T cells expressing α-syn140-YFP fusion proteins harboring one of the following mutations: (a) A30G, (b) E46K, (c) K80E, (d) G51D, (e) A53E, (f) A53T, (g) A53V, (h) V55Y, (i) V66F, or (j) V74P. Quantification of α-synuclein prion infectivity (× 10^5^ arbitrary units [A.U.]) shown as mean ± SD. * = *P* < 0.05.

We next evaluated the biochemical profile of A53T PFFs before and after mouse passage. The A53T PFF inoculum was partially resistant to 12.5 μg/mL PK, but this resistance fell to 6.25 μg/mL after transmission to TgM83^+/-^ mice (Fig 6C), indicative of a change in strain stability. Similarly, the overall resistance of A53T PFFs to denaturation by GdnHCl also decreased after passage in mice (Fig 6D and 6E). Together, these findings indicate that passage in TgM83^+/-^ mice either alters the strain stability of A53T PFFs or selects for a subset of the total fibril conformations from those present in the initial pool of PFF structures. To determine which of these two possibilities occurred, we compared infectivity profiles for the A53T PFFs before and after passage in our panel of α-syn140-YFP cell lines. Consistent with previous findings [8], the A53T PFFs robustly infected all 10 cell lines tested, regardless of mutation (*P* < 0.05; S5 Fig and S4 Table). However, this infectivity profile changed after passage in the TgM83^+/-^ mice (Fig 7 and S5 Table). While the mouse-passaged A53T PFF samples were able to significantly infect cells expressing the A30G, G51D, A53T, A53V, and V55Y mutations (*P* < 0.05; Fig 7 and S3 Fig), the infection in the A30G and G51D lines was sparse and below our cutoff for biological significance. Notably, the passaged A53T PFF samples were no longer able to replicate in cell lines expressing the E46K (*P* = 0.63), K80E (*P* = 0.29), A53E (*P* = 0.13), V66F (*P* = 0.24), or V74P mutations (*P* = 0.12; Fig 7 and S3 Fig). This shift in infectivity profile suggests that among the many conformations compatible with each cell line present in the starting PFF pool, only a subset were present following passage in TgM83^+/-^ mice, consistent with our hypothesis that mouse passage of PFFs results in strain selection.

## Discussion

Variation in clinical presentation and disease progression between synucleinopathy patients is hypothesized to be due to differences in α-synuclein strains. As previously discussed, disease in a subset of PAF patients begins in the periphery before phenoconverting into central dysfunction, seen as either MSA or LBD. It remains unknown if and how a peripheral disease origin impacts disease pathogenesis across α-synuclein strains, or if strain adaptation or selection during neuroinvasion leads to a change in strain biology. Understanding the role of strain adaptation in disease, particularly in patients with an initial peripheral presentation, would help better predict the progression of disease and symptom onset in PAF patients. Addressing this question in the studies reported here, we investigated the effect of neuroinvasion on α-synuclein strain biology, compared to a central onset of disease, as well the potential for strain selection to alter biochemical and biological properties of α-synuclein prions.

We first investigated the effect of inoculation route on α-synuclein strain biology, given that neuroinvasion is known to impact PrP^Sc^ strain stability [26–28]. Our results indicate that neuroinvasion of MSA prions inoculated into TgM83^+/-^ mice occurs via retrograde spread through the spinal cord, similar to what has been shown previously with α-synuclein PFFs [43] and PrP^Sc^ prions [44]. Interestingly, the density of phosphorylated α-synuclein neuropathology in the brain was lower in the sc.n.-inoculated mice compared to i.c.-inoculated animals, though this difference was not significant (Fig 1B). Given that expression of the α-synuclein transgene is ~6-fold higher in the spinal cord compared to the brain in TgM83^+/-^ mice [45], we hypothesize that this difference in pathology accumulation is the result of extensive lesions within the spinal cord of sc.n.-inoculated mice accelerating the progressive onset of motor signs before substantial brain accumulation can occur. Notably, we also found robust bilateral pathology in the spinal cord, indicating that α-synuclein prions likely spread to contralateral neural tracts early in neuroinvasion (Fig 2B), though the lack of detectable neuropathology in the spinal cord prior to ~60% of the incubation period or later suggests spreading occurs prior to the formation of mature inclusions (Table 1). Out of the two animals positive for α-synuclein deposition in the spinal cord at the 60% timepoint, only one brain was positive for α-synuclein prions using our cell assay (Fig 3B). Together, these data suggest that neuroinvasion can occur as early as 60% of the MSA incubation, and that future studies should investigate additional timepoints (i.e., 70% or 80%) to adequately resolve the rate of neuroinvasion.

We previously used cell infectivity studies to investigate the maintenance of MSA strain properties after passage in three different Tg mouse models, finding no change before or after i.c. inoculation [9,46]. However, these findings were limited to the use of cell lines expressing either the A30P, E46K, or A53T mutations. The recent expansion of our panel of α-syn140-YFP cell lines to interrogate MSA strain properties showed that the A30G, A53E, V55Y, and K80E mutations block MSA replication *in vitro* [30]. Using this same panel of mutant cells here to investigate strain properties after both primary passage (inoculum) and secondary passage (i.c.- and sc.n.-inoculated cohorts) in TgM83^+/-^ mice enabled our important findings in support of the hypothesis that passaging MSA *in vivo* alters strain properties, independent of inoculation route (Table 2). Notably, after primary mouse-passage, MSA16 prions were no longer able to replicate in cells expressing the A30G, G51D, V55Y, V66F, and V74P mutations, providing strong evidence that mouse-passaged MSA prions exhibit distinct biological properties from the MSA prions isolated from human patient samples. Moreover, this altered infectivity profile remains stable after secondary passage. Given the location of residue A53 at the protofilament interface in all known MSA α-synuclein cryo-EM structures [1,3], we hypothesize that this observed strain adaptation occurs due to deformed templating, which has been postulated to occur in PrP^Sc^ prions [32,47–49]. Here, we anticipate that the α-synuclein conformation in MSA must adopt a slightly altered structural arrangement to accommodate the A53T mutation present in the TgM83^+/-^ mouse model, resulting in a similar, but unique, α-synuclein fibril structure. It should be noted that despite these important biological differences, the biochemical properties assessed by PK digest and GdnHCl denaturation were consistent with the starting MSA inoculum, as previously reported [8], demonstrating the superior power of the α-syn140-YFP cell assay panel to resolve strain differences for α-synuclein prions compared to traditional strain typing methods.

In reporting these important advances, we also recognize important caveats to our findings. First, we recognize that some of the mutations tested here are associated with LBDs whereas others are novel. It should be noted that given our assay design, these mutations do not impact strain formation, and should only be considered experimental tools used to investigate strain properties. Additionally, differences in sample (presence of posttranslational modifications, fibril size, etc.) may impact uptake and infectivity. To circumvent the potential confounding effects of differences in uptake, we use Lipofectamine 2000 to facilitate cellular uptake in our assay. This allows us to interpret a negative result as an inability to replicate in a particular cell line independently from impaired cellular uptake. We also recognize that the rate of cell division may also greatly impact protein misfolding. Given that HEK293T cells double roughly every 24 h, α-synuclein strains with slower replication kinetics may be cleared from the cell prior to mounting an infection, which would favor detection of strains with faster replication kinetics in our assay. Lastly, while we endeavor to develop monoclonal cell lines with similar levels of α-synuclein fusion protein expression, it is impossible to precisely control for expression differences, which may ultimately influence infection levels between cells. As such, the fluorescence/cell measurements made from one cell line should not be directly compared with measurements made from a second cell line.

In addition to our studies unexpectedly showing evidence of MSA strain adaptation in the TgM83^+/-^ mouse model, it is also important to recognize the implications of our findings that MSA strain properties are unaffected by inoculation route. This result suggests that in patients with PAF who later phenoconvert to MSA, the α-synuclein strain present in the periphery is likely the MSA strain, rather than a different PAF-specific α-synuclein conformation that adapts upon neuroinvasion and phenoconversion to MSA. However, studies testing strain properties using samples collected from PAF patients prior to phenoconversion are needed to definitively address this question. While no structural, biochemical, or biological analyses are currently available for α-synuclein fibrils in PAF patients, the difference in clinical phenotype and disease progression suggests there may be strain differences that contribute to determining whether or not a patient develops a central synucleinopathy. As a result, our findings suggest that diagnostic assays capable of discriminating between α-synuclein strains in the periphery may be capable of successfully differentiating a true PAF patient from one who will ultimately phenoconvert to MSA or LBD. In this way, by better understanding the peripheral α-synuclein prion properties associated with each disease, we may be closer to improving diagnosis and predicting disease progression for synucleinopathy patients.

In addition to assessing the potential for α-synuclein adaptation during neuroinvasion, we investigated the role of conformational selection of α-synuclein PFFs during i.c. transmission in mice on strain properties. In previous studies using wild-type and E46K PFFs inoculated in the TgM20^+/-^ and TgM47^+/-^ mice, which express wild-type and E46K α-synuclein, respectively, cell assay data suggested the potential for selection of a sub-population of α-synuclein conformations during passage [7,9]. Using our larger panel of α-syn140-YFP cell lines here, we observed universal infection with the A53T PFFs prior to mouse passage (S5 Fig). In comparison, mouse-passaged A53T PFFs were unable to replicate in cells expressing the A30G, E46K, K80E, G51D, A53E, V66F, and V74P mutations (Fig 7). In these experiments, both the inoculum and the mice (e.g., agent and host) express the same A53T mutation, suggesting deformed templating is less likely to contribute to this observed outcome. Instead, consistent with the cloud hypothesis [50–52], the selection of a sub-population from among the larger A53T PFF conformations due to a change in the replication environment is the more likely explanation for the altered biological properties measured here.

These findings provide critical insight into the use of PFFs for research studies and drug development. PFFs are widely used as a proxy for human tissue, despite the fact that they adopt misfolded conformations that often differ drastically from the fibril conformations that are present in human patient samples [1–3,53–61]. Importantly, efforts to amplify disease-relevant α-synuclein structures using an RT-QuIC assay have also failed to replicate the fibril conformations present in synucleinopathy patient samples [14,62,63]. These findings are consistent with our cell assay data showing that the infectivity profile of mouse-passaged A53T PFFs is distinct from the MSA strain profile, both before and after mouse-passage (Table 2). Most notably, differences in the ability to replicate in the α-syn140*V55Y-YFP cell line point to unique biological properties between the two strains. While it is currently unknown how different the mouse-passaged MSA and A53T PFF structures are, the differences in biological and biochemical properties reported here add to the growing recognition that PFFs are not currently a suitable model for investigating α-synuclein strain biology in human disease [7,29].

In summary, in the studies reported here, we paired animal bioassay with biochemistry, neuropathology, and *in vitro* models of α-synuclein misfolding to directly investigate adaptation and selection of two α-synuclein strains. We provide the first evidence that MSA prions undergo strain adaptation during passage in TgM83^+/-^ mice, though these changes in strain properties are unaltered by neuroinvasion. This unexpected finding, which we could only resolve via cell infectivity profile, suggests that deformed templating contributes to structural rearrangement of α-synuclein strains when host and agent genotype mismatch occurs. Additional studies are needed to determine if this same phenomenon can impact replication of α-synuclein fibrils in LBD patients. Additionally, we used our expanded panel of α-syn140-YFP cell lines to show that i.c. inoculation of A53T PFFs into TgM83^+/-^ mice results in selection of a sub-population of fibril conformations. More importantly, these studies generated important data underscoring the biological differences between PFFs and MSA prions, highlighting the need to standardize how investigators select which α-synuclein strain is used for small molecule screens and diagnostic development to increase the likelihood of successful clinical translation. Combined, these data provide direct evidence of α-synuclein prion adaptation and selection, while also establishing our cellular assay as a powerful, high-resolution strain typing tool.

## Materials and Methods

### Ethics statement

Animals were maintained in compliance with the *Guide for the Care and Use of Laboratory Animals*. All procedures used in this study were approved by the University of Massachusetts Amherst Institutional Animal Care and Use Committee (IACUC). Frozen brain tissue samples from one control (C2) and one MSA (MSA16) patient sample were provided by the Massachusetts Alzheimer’s Disease Research Center. University of Massachusetts Amherst and Colorado State University Institutional Review Board approvals were not required for this study. Both human samples were anonymized.

### Mice

Animals were housed in ABSL-2 conditions with a 12-hour light/dark cycle with free access to food and water. TgM83^+/-^ mice were generated by breeding TgM83^+/+^ mice [45] with B6C3F1 mice purchased from Jackson Laboratory. All mice were group housed unless health concerns deemed individual housing necessary.

### Inoculations

Fresh-frozen mouse tissue was homogenized in calcium- and magnesium-free 1× Dulbecco’s phosphate buffered saline (DPBS) using the Omni Tissue Homogenizer (Omni International) to create a 10% (wt/vol) homogenate. Homogenates were diluted to 5 mg/mL in DPBS. Recombinant A53T human α-synuclein (rPeptide) was fibrillized in 1× DPBS as previously described [8] before diluting in 1× DPBS to a final concentration of 1 mg/mL.

For intracranial inoculations, 6-to-7-week-old TgM83^+/-^ mice were anesthetized with isoflurane. The i.c. injections were performed using 20 μL of 5 mg/mL brain homogenate or 1 mg/mL fibrils into the thalamus. For sc.n. injections, 12-week-old TgM83^+/-^ mice were anesthetized with isoflurane before injection as previously described [44]. Briefly, hair was removed from the left hindlimb before making a small incision (≤1 cm) to expose the sciatic nerve parallel to the femur. A 28-gauge needle was moved up and down parallel to the nerve and 20 μL of either 5 mg/mL brain homogenate or 1 mg/mL fibrils were slowly injected. The incision was closed using vicryl rapide suture. After inoculation, all mice were assessed 3 times each week for the onset of neurological signs based on standard prion disease criteria [64]. Mice injected with mouse-passaged MSA or PFFs were euthanized following the onset of progressive neurological signs. Control-injected mice were euthanized at 365 dpi (for i.c. inoculations) or 500 dpi (for sc.n. inoculations). Following euthanasia, the brain was removed and bisected down the midline. One half was fixed in formalin for neuropathological assessment and the other half was frozen for biochemical and biological analyses. The spinal column from sc.n.-injected mice was collected in formalin for pathological assessment.

### Immunohistochemistry and neuropathology

Formalin-fixed mouse half-brains were cut into four coronal sections prior to processing through graded alcohols, xylene, paraffin, and embedding, as previously described [9]. The tissue was cut into 8 μm sections, mounted on slides, deparaffinized, and underwent heat-mediated antigen retrieval with citrate buffer (0.1 *M*, pH 6) for 20 min. Slides were incubated overnight at room temperature in primary antibodies EP1536Y (1:1,000; Abcam) and glial fibrillary acidic protein (GFAP; 1:500; Abcam) after blocking in 10% (vol/vol) normal goat serum. Secondary antibodies conjugated to AlexaFluor 488 and 594 (1:500; ThermoFisher) were used to detect primary antibody staining. Hoechst 33342 (1:5,000; ThermoFisher) was used to label nuclei. Spinal columns were decalcified in Rapid Bone Decalcifier and cut into 5 mm thick sections before being processed as described above. Spinal column tissue was cut into 10 μm sections before preparation and staining as described above. Slides were imaged using the Lionheart FX automated microscope using the 10x objective and analyzed using the BioTek Gen5 software package. A-synuclein pathology was quantified using a pixel intensity threshold set using positive and negative control slides, and regions of interest were drawn around the caudoputamen (Cd), piriform cortex & amygdala (PC), hippocampus (HC), thalamus (Thal), HTH, Mid, and pons. The percentage of positive pixels in each region was averaged across mice in each experimental group. Spinal cord pathology was qualitatively assessed by the presence or absence of disease-associated α-synuclein phosphorylated at serine 129 in the cervical, thoracic, lumbar, and sacral regions of the spinal cord. Representative images of phosphorylated *α*-synuclein pathology in the brain and spinal cord were captured using the Leica DMi8 inverted fluorescent microscope and processed using the accompanying LAS X software.

### Α-synuclein prion quantification assay

The frozen brain tissue was homogenized to 10% (wt/vol) in 1× DPBS as described above. Pathogenic protein aggregates were isolated from the brain homogenates using phosphotungstic acid (PTA; Sigma), as described previously [16,65]. The isolated protein pellets were diluted in 1× DPBS [A30G (1:5), E46K (1:10), G51D (1:4), A53E (1:10), A53T (1:14), A53V (1:7), V55Y (1:20), V66F (1:10), V74P (1:10), and K80E (1:20)] before testing in HEK293T cells expressing mutant human α-synuclein fused to YFP using conditions reported previously [30]. A53T PFFs were diluted in 1× DPBS to 25 μg/mL before infecting HEK293T cells. Each sample was plated in 6 technical replicate wells prior to incubation for 4 d. The Lionheart FX automated fluorescent microscope (Agilent BioTek) was used to collect DAPI and YFP images from 4 regions of interest in each well. Custom algorithms were built using BioTek Gen5 software to quantify intracellular aggregates in living cells, represented as total fluorescence per cell (× 10^5^, arbitrary units, A.U.) using a fluorescence and size threshold. The A53E and A53V cell lines had separate algorithms for analyzing the A53T PFF inoculum and the mouse-passaged samples due to large differences in aggregate morphology. Values from all 4 regions were combined to determine 1 value for each well, and values from the 6 technical replicate wells were combined to determine an average measure for each sample.

### Proteinase K digestion

Detergent extracted brain homogenates (1 mg/mL) or PFFs (200 μg/mL) were digested with PK as we previously reported [7,8]. Briefly, extracted brain homogenate or PFFs were incubated with PK (0, 1.25, 2.5, 6.25, 12.5 μg/mL) at 37 °C while shaking for 30 min. The reaction was stopped with 5 mM PMSF, and samples were centrifuged at 100,000 × *g* for 1 h at 4 °C. The resulting protein pellets were resuspended in 50 μL 1× NuPAGE LDS loading buffer containing β-mercaptoethanol then boiled for 10 min before immunoblotting.

### Guanidine hydrochloride denaturation

Brain homogenates were extracted 9:1 (vol:vol) in 10× detergent buffer (5% [vol/vol] Nonidet P-40, 5% [wt/vol] sodium deoxycholate [DOC] in DPBS) on ice for 20 min. Samples were centrifuged for 5 min at 1000 × *g*, and the supernatant (20 μL) or diluted PFFs (200 μg/mL) were incubated with GdnHCl, as we described previously [7,8]. Briefly, samples were incubated with 0, 1, 1.5, 2, 2.5, 3 *M* GdnHCl for 2 h at 22 °C with shaking. The GdnHCl concentration was adjusted to 0.4 *M* GdnHCl, and samples were centrifuged at 4 °C for 1 h at 100,000 × *g*. The final pellets were resuspended in 40 μL 1× NuPAGE LDS loading buffer with β-mercaptoethanol. Samples were boiled for 10 min before immunoblotting.

### Immunoblotting

PK-digested and GdnHCl-denatured samples (20 μL for brain homogenates and 5 μL for A53T PFFs) were loaded onto a 10% Bis-Tris gel (ThermoFisher), and SDS-PAGE was performed using the MES buffer system followed by protein transfer to a polyvinylidene fluoride (PVDF) membrane. After transfer, the membrane was fixed in 0.4% formalin for 30 min at room temperature and then incubated for a further 30 min in blocking buffer (5% [wt/vol] nonfat milk in 1× Tris-buffered saline and 0.05% [vol/vol] Tween 20) at room temperature. Blots were probed with primary antibody for either phosphorylated α-synuclein (EP1536Y; 1:4,000; Abcam) or total α-synuclein (MJFR1; 1:12,500; Abcam) in blocking buffer overnight at 4°C in a heat-sealed pouch. Membranes were washed in 1× TBST three times and then incubated for 1 h with goat anti-rabbit secondary antibody conjugated to horseradish peroxidase (1:10,000; Abcam) diluted in blocking buffer. After incubation, membranes were again washed three times in 1× TBST before imaging using the Azure 600 (Azure Biosystems).

### Statistical analysis

Data were analyzed using GraphPad Prism software (version 10) and are presented as mean ± standard deviation. Kaplan–Meier curves of incubation periods were analyzed using a log-rank Mantel–Cox test. Quantification of neuropathology was analyzed using a two-way ANOVA with Šídák’s multiple comparisons test. Cell assay data comparing human patient samples (C2 and MSA16) and mouse-passaged inocula were analyzed using a one-way ANOVA with Tukey’s multiple comparisons post hoc test. Cell assay data comparing i.c.- and sc.n.-injected control and MSA samples from terminal mice were analyzed using a two-way ANOVA with Tukey’s multiple comparisons post hoc test. Finally, cell assay data comparing passaged control inocula with A53T PFFs, both the starting inocula as well as following mouse passage, were analyzed using an unpaired t-test with Welch’s correction. Significance was determined with a *P* value < 0.05.

## Supporting information

S1 Fig Intracranial injection of passaged MSA induces more robust phosphorylated α-synuclein pathology compared to sciatic nerve injection. TgM83^+/-^ mice were inoculated with 20 μL of brain homogenate (standardized to 5 mg/mL total protein) from either mouse-passaged control (top) or MSA patient samples (bottom). Mice were injected either intracranially (left) or into the sciatic nerve (right). Representative images of the pons show phosphorylated α-synuclein (EP1536Y; 1:1,000) in green, glial fibrillary acidic protein (GFAP; 1:500) in red, and DAPI in blue. Scale bar, 200 μm.
(TIF)

S2 Fig Human patient MSA and mouse-passaged MSA inoculum selectively infect α-syn140-YFP cells. Α-synuclein prions were isolated from one control (C2, light gray) or one MSA patient sample (MSA16, dark blue), as well as the mouse-passaged control (dark gray) and MSA patient samples (light blue), which were used as inocula, via phosphotungstic acid precipitation. Resulting pellets were tested for infectivity in HEK293T cells expressing α-syn140-YFP fusion proteins harboring one of the following mutations: (a) A30G, (b) E46K, (c) K80E, (d) G51D, (e) A53E, (f) A53T, (g) A53V, (h) V55Y, (i) V66F, or (j) V74P. Quantification of α-synuclein prion infectivity (× 10^5^ arbitrary units [A.U.]) shown as mean ± SD. * = *P* < 0.05.
(TIF)

S3 Fig Representative images of α-synuclein prion infection in α-syn140-YFP cell lines. TgM83^+/-^ mice were inoculated with 20 μL of brain homogenate (standardized to 5 mg/mL total protein) of mouse-passaged control or MSA patient sample into the sciatic nerve (sc.n.) or intracranially with 1 mg/mL human α-synuclein preformed fibrils (PFFs) with the PD-associated A53T mutation. Frozen half-brains collected from terminal mice were homogenized in 1× DPBS. Phosphotungstic acid was used to precipitate α-synuclein prions, and the resulting pellets were incubated with HEK293T cells expressing α-syn140-YFP fusion proteins harboring the following mutations: A30G, E46K, K80E, G51D, A53E, A53T, A53V, V55Y, V66F, or V74P. Hoechst stain is shown in blue, YFP in yellow. Scale bar, 60 μm.
(TIF)

S4 Fig A53T PFFs induce robust α-synuclein pathology in TgM83^+/-^ mice. TgM83^+/-^ mice were intracranially (i.c.) inoculated with 20 μL of brain homogenate (standardized to 5 mg/mL total protein) of mouse-passaged control patient sample (left) or 1 mg/mL human α-synuclein PFFs with the PD-associated A53T mutation (right). Half-brains were collected from terminal mice in formalin followed by processing, embedding, and coronal sectioning. Representative images of the pons from either control- or A53T PFF-injected mice show phosphorylated α-synuclein (EP1536Y, 1:1,000) in green, glial fibrillary acidic protein (GFAP, 1:500) in red, and DAPI in blue. Scale bar, 200 μm.
(TIF)

S5 Fig A53T PFFs uniformly infect mutant α-syn140-YFP cell lines. Recombinant human α-synuclein with the A53T mutation was fibrillized in 1× DPBS and diluted to 25 μg/mL in 1× DPBS for cell infection. Mouse-passaged control sample (gray) or A53T preformed fibrils (PFFs; green) were incubated with HEK293T cells expressing α-syn140-YFP fusion proteins harboring one of the following mutations: (a) A30G, (b) E46K, (c) K80E, (d) G51D, (e) A53E, (f) A53T, (g) A53V, (h) V55Y, (i) V66F, or (j) V74P. Quantification of α-synuclein prion infectivity (× 10^5^ arbitrary units [A.U.]). Data shown as mean ± SD. * = *P* < 0.05.
(TIF)

S1 Table Infectivity of time course studies from secondary passage of MSA inoculated via sciatic nerve in α-syn140*A53T-YFP. (DOCX)

S2 Table Infectivity of human patient samples and primary passage of human patient samples in cultured cells. (DOCX)

S3 Table Infectivity of secondary passage of MSA patient samples intracranially and via sciatic nerve injection in cultured cells. (DOCX)

S4 Table Infectivity of recombinant A53T preformed fibrils in cultured cells. (DOCX)

S5 Table Infectivity of mouse-passaged recombinant A53T preformed fibrils in cultured cells. (DOCX)
