# Supplementary figures and images for: A-synuclein prion strains differentially adapt after passage in mice
Source: PLoS Pathog. 2024 Dec 6;20(12):e1012746. doi: 10.1371/journal.ppat.1012746 (PMC11623799; doi:10.1371/journal.ppat.1012746)

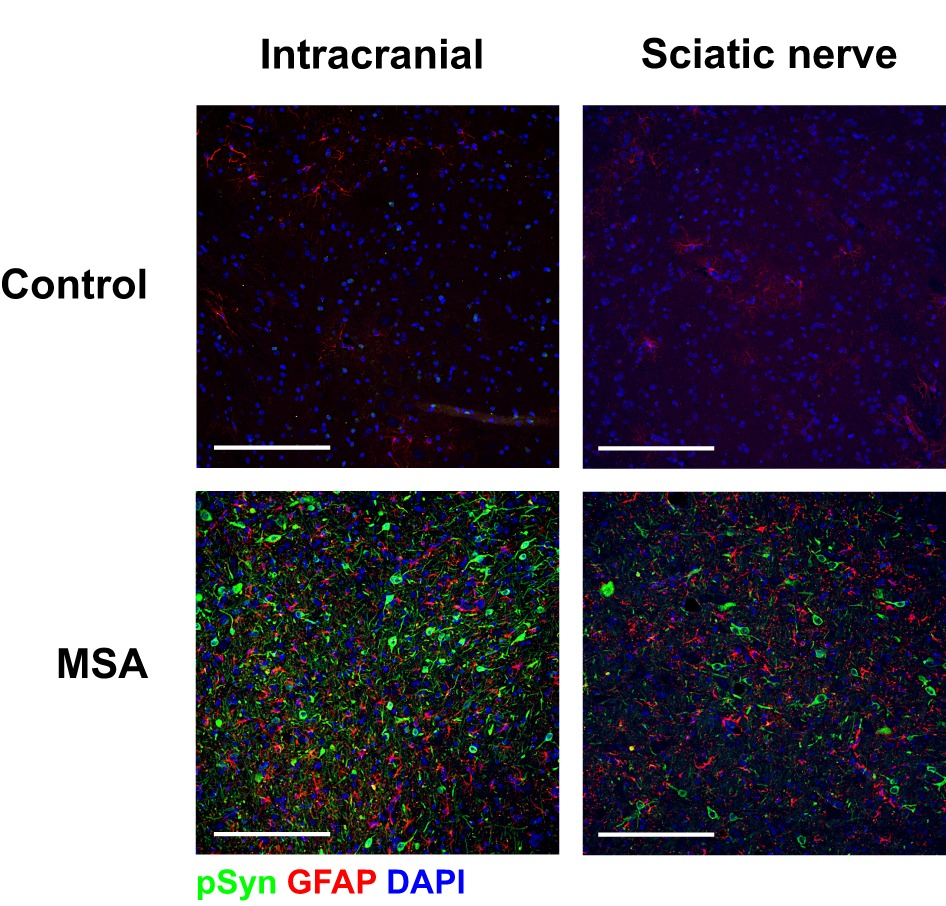

Supplement: S1 Fig — TgM83+/- mice were inoculated with 20 μL of brain homogenate (standardized to 5 mg/mL total protein) from either mouse-passaged control (top) or MSA patient samples (bottom). Mice were injected either intracranially (left) or into the sciatic nerve (right). Representative images of the pons show phosphorylated α-synuclein (EP1536Y; 1:1,000) in green, glial fibrillary acidic protein (GFAP; 1:500) in red, and DAPI in blue. Scale bar, 200 μm. (TIF) [file ppat.1012746.s001.tif]

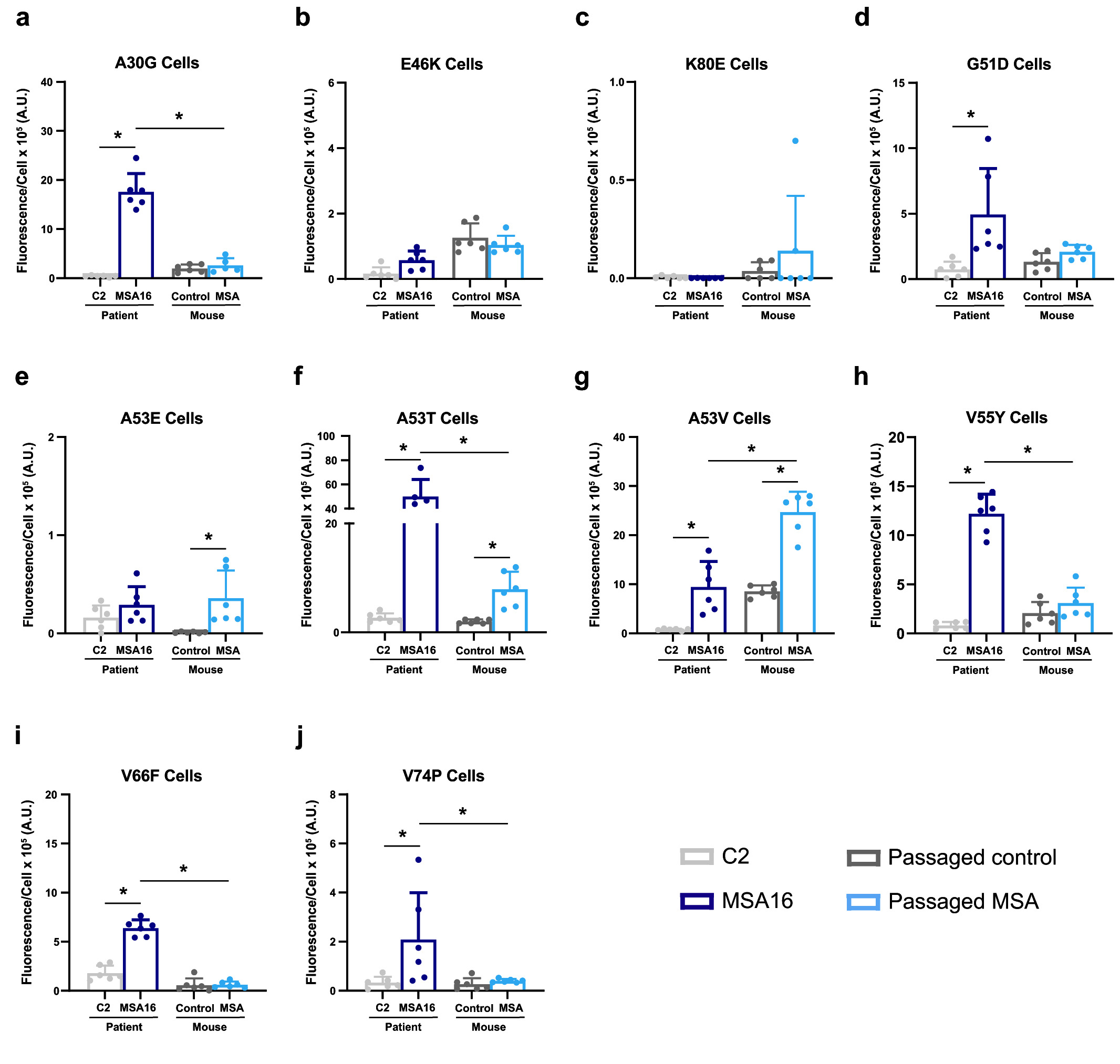

Supplement: S2 Fig — Α-synuclein prions were isolated from one control (C2, light gray) or one MSA patient sample (MSA16, dark blue), as well as the mouse-passaged control (dark gray) and MSA patient samples (light blue), which were used as inocula, via phosphotungstic acid precipitation. Resulting pellets were tested for infectivity in HEK293T cells expressing α-syn140-YFP fusion proteins harboring one of the following mutations: (a) A30G, (b) E46K, (c) K80E, (d) G51D, (e) A53E, (f) A53T, (g) A53V, (h) V55Y, (i) V66F, or (j) V74P. Quantification of α-synuclein prion infectivity (× 105 arbitrary units [A.U.]) shown as mean ± SD. * = P < 0.05. (TIF) [file ppat.1012746.s002.tif]

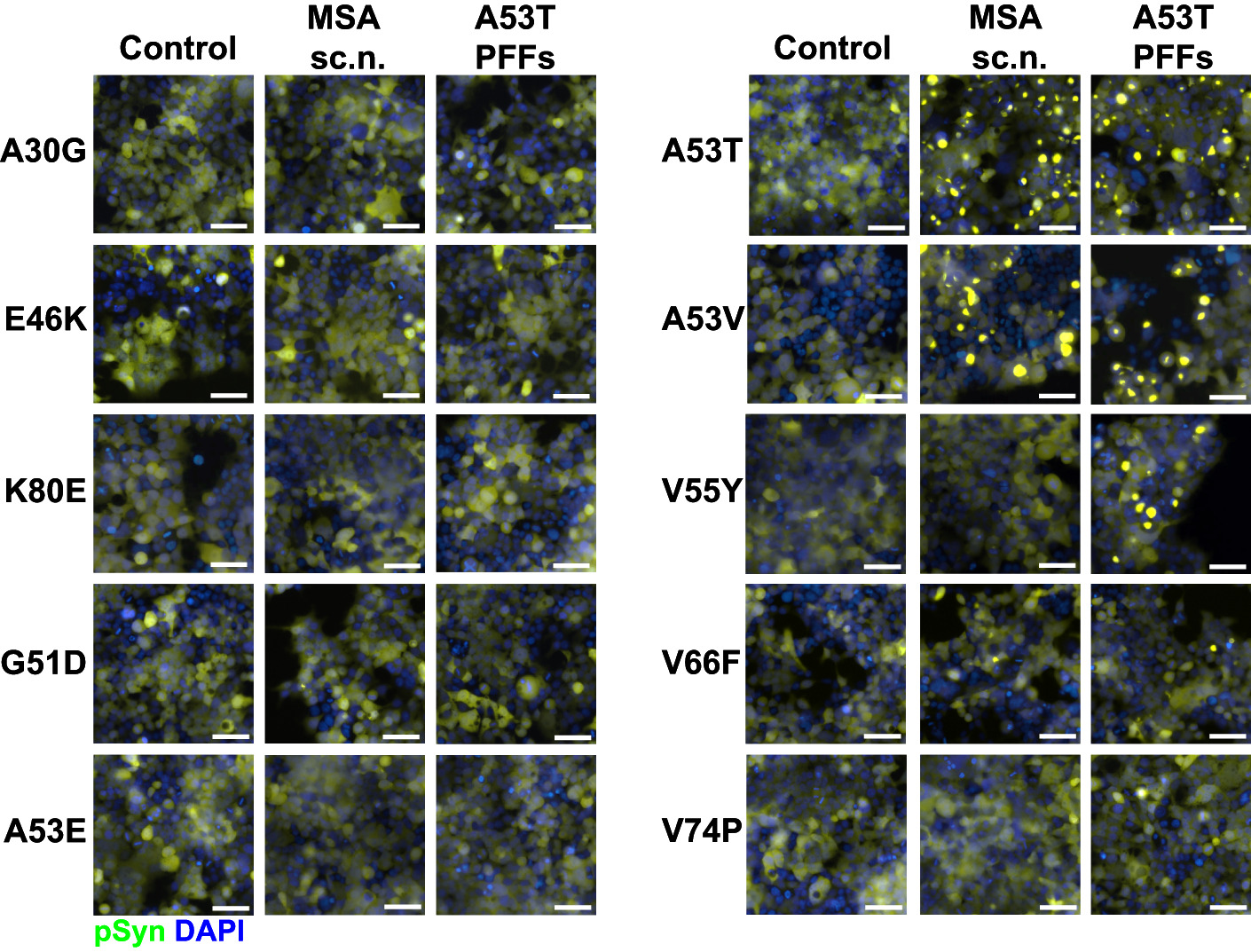

Supplement: S3 Fig — TgM83+/- mice were inoculated with 20 μL of brain homogenate (standardized to 5 mg/mL total protein) of mouse-passaged control or MSA patient sample into the sciatic nerve (sc.n.) or intracranially with 1 mg/mL human α-synuclein preformed fibrils (PFFs) with the PD-associated A53T mutation. Frozen half-brains collected from terminal mice were homogenized in 1× DPBS. Phosphotungstic acid was used to precipitate α-synuclein prions, and the resulting pellets were incubated with HEK293T cells expressing α-syn140-YFP fusion proteins harboring the following mutations: A30G, E46K, K80E, G51D, A53E, A53T, A53V, V55Y, V66F, or V74P. Hoechst stain is shown in blue, YFP in yellow. Scale bar, 60 μm. (TIF) [file ppat.1012746.s003.tif]

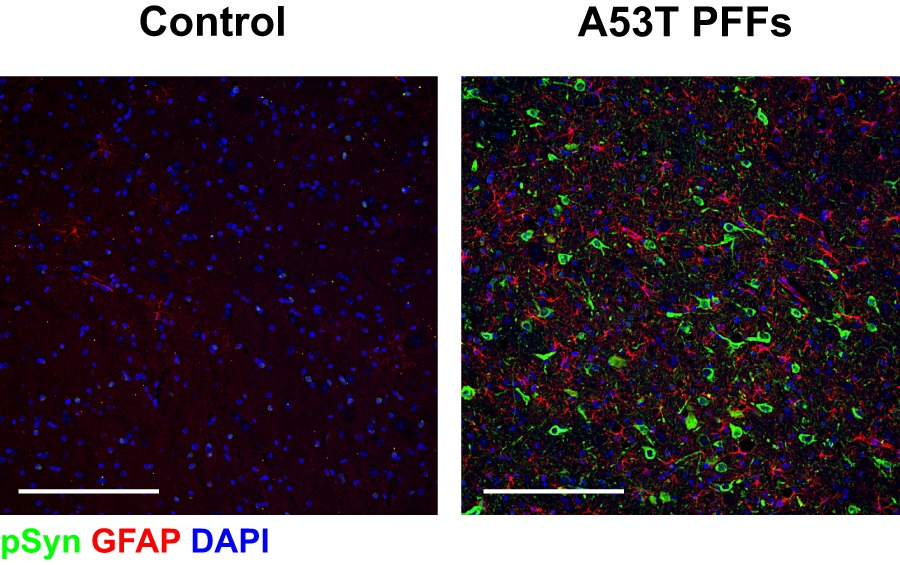

Supplement: S4 Fig — TgM83+/- mice were intracranially (i.c.) inoculated with 20 μL of brain homogenate (standardized to 5 mg/mL total protein) of mouse-passaged control patient sample (left) or 1 mg/mL human α-synuclein PFFs with the PD-associated A53T mutation (right). Half-brains were collected from terminal mice in formalin followed by processing, embedding, and coronal sectioning. Representative images of the pons from either control- or A53T PFF-injected mice show phosphorylated α-synuclein (EP1536Y, 1:1,000) in green, glial fibrillary acidic protein (GFAP, 1:500) in red, and DAPI in blue. Scale bar, 200 μm. (TIF) [file ppat.1012746.s004.tif]

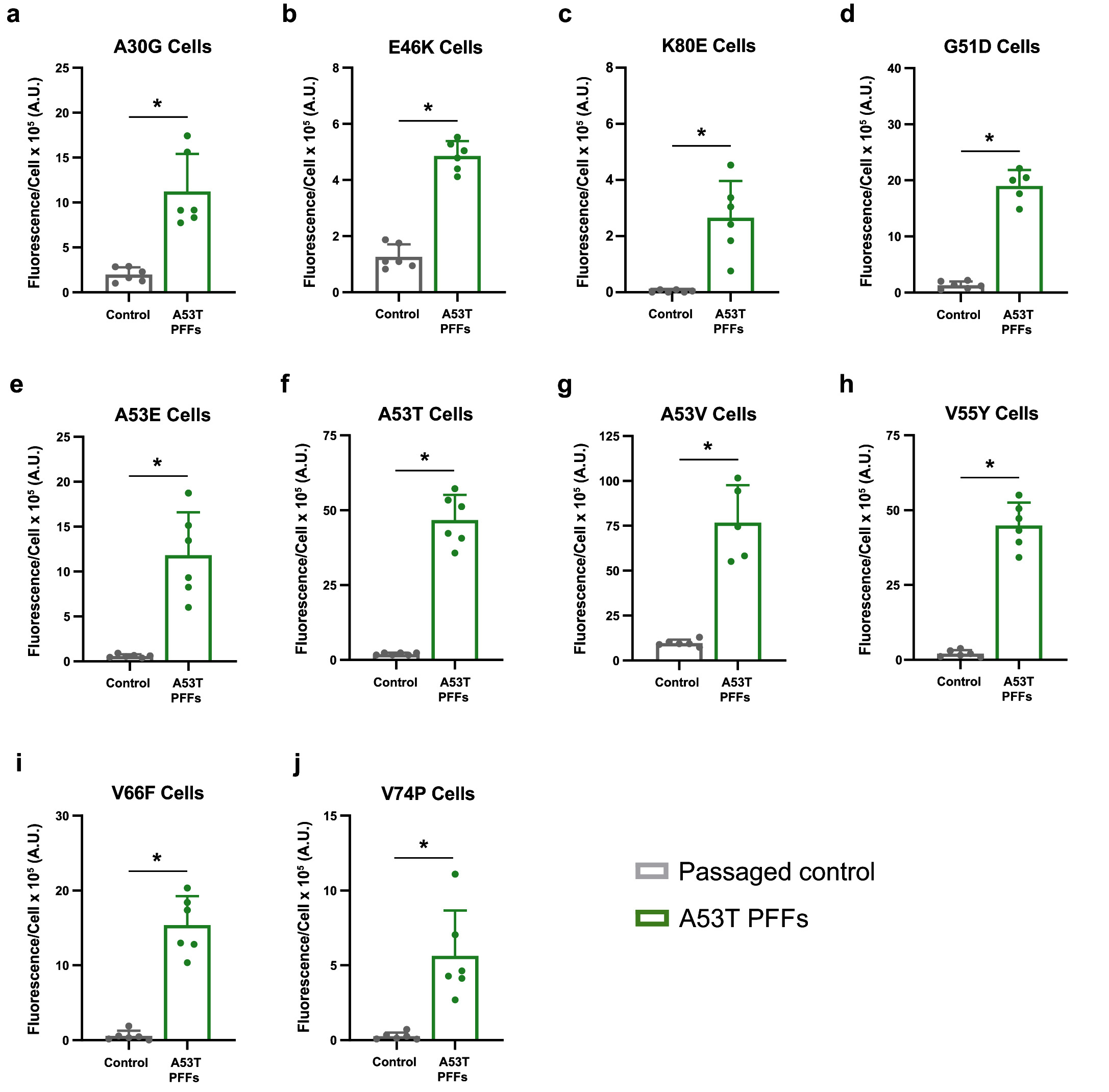

Supplement: S5 Fig — Recombinant human α-synuclein with the A53T mutation was fibrillized in 1× DPBS and diluted to 25 μg/mL in 1× DPBS for cell infection. Mouse-passaged control sample (gray) or A53T preformed fibrils (PFFs; green) were incubated with HEK293T cells expressing α-syn140-YFP fusion proteins harboring one of the following mutations: (a) A30G, (b) E46K, (c) K80E, (d) G51D, (e) A53E, (f) A53T, (g) A53V, (h) V55Y, (i) V66F, or (j) V74P. Quantification of α-synuclein prion infectivity (× 105 arbitrary units [A.U.]). Data shown as mean ± SD. * = P < 0.05. (TIF) [file ppat.1012746.s005.tif]
