# Supplementary material for: A-synuclein prion strains differentially adapt after passage in mice
Source: PLoS Pathog. 2024 Dec 6;20(12):e1012746. doi: 10.1371/journal.ppat.1012746 (PMC11623799; doi:10.1371/journal.ppat.1012746)
Supplement: S1 Table — (DOCX) [file ppat.1012746.s006.docx]

**S1 Table. Infectivity of time course studies from secondary passage of MSA inoculated via sciatic nerve in α-syn140*A53T-YFP cells.**

| **Control** | **30% MSA** | **45% MSA** | **60% MSA** | **100% MSA** |
| --- | --- | --- | --- | --- |
| 1.8 ± 0.7 | 1.1 ± 0.1 | 1.4 ± 0.4 | 4.1 ± 4.8 | 15 ± 6.5 |

*Data reported as mean cell infection ± standard deviation.*
