# Supplementary material for: A-synuclein prion strains differentially adapt after passage in mice
Source: PLoS Pathog. 2024 Dec 6;20(12):e1012746. doi: 10.1371/journal.ppat.1012746 (PMC11623799; doi:10.1371/journal.ppat.1012746)
Supplement: S2 Table — (DOCX) [file ppat.1012746.s007.docx]

**S2 Table. Infectivity of human patient samples and primary passage of human patient samples in cultured cells.**

| **Cell Line** | **C2** | **MSA16** | **Passaged control** | **Passaged MSA** |
| --- | --- | --- | --- | --- |
| A30G | 0.4 ± 0.2 | 18 ± 3.7 | 2.0 ± 0.8 | 2.6 ± 1.5 |
| E46K | 0.2 ± 0.2 | 0.6 ± 0.3 | 1.3 ± 0.4 | 1.0 ± 0.3 |
| K80E | 0.0 ± 0.0 | 0.0 ± 0.0 | 0.0 ± 0.0 | 0.1 ± 0.3 |
| G51D | 0.7 ± 0.6 | 4.9 ± 3.5 | 1.3 ± 0.7 | 2.1 ± 0.5 |
| A53E | 0.2 ± 0.1 | 0.3 ± 0.2 | 0.0 ± 0.0 | 0.4 ± 0.3 |
| A53T | 2.6 ± 0.9 | 50 ± 14 | 2.0 ± 0.4 | 7.9 ± 3.2 |
| A53V | 0.3 ± 0.1 | 9.6 ± 5.1 | 8.6 ± 1.2 | 25 ± 4.1 |
| V55Y | 0.8 ± 0.4 | 12 ± 2.0 | 2.1 ± 1.1 | 3.1 ± 1.6 |
| V66F | 1.8 ± 0.8 | 6.4 ± 0.8 | 0.6 ± 0.7 | 0.6 ± 0.3 |
| V74P | 0.3 ± 0.2 | 2.1 ± 1.9 | 0.3 ± 0.2 | 0.4 ± 0.1 |

*Data reported as mean cell infection ± standard deviation.*
