# Supplementary material for: A-synuclein prion strains differentially adapt after passage in mice
Source: PLoS Pathog. 2024 Dec 6;20(12):e1012746. doi: 10.1371/journal.ppat.1012746 (PMC11623799; doi:10.1371/journal.ppat.1012746)
Supplement: S3 Table — (DOCX) [file ppat.1012746.s008.docx]

**S3 Table. Infectivity of secondary passage of MSA patient samples intracranially and via sciatic nerve injection in cultured cells.**

| **Cell Line** | **Control i.c.** | **MSA i.c.** | **Control sc.n.** | **MSA sc.n.** |
| --- | --- | --- | --- | --- |
| A30G | 2.0 ± 0.4 | 2.4 ± 0.9 | 1.6 ± 0.2 | 2.4 ± 0.5 |
| E46K | 1.1 ± 0.6 | 1.0 ± 0.3 | 0.8 ± 0.2 | 1.3 ± 0.7 |
| K80E | 0.1 ± 0.1 | 0.1 ± 0.1 | 0.1 ± 0.1 | 0.1 ± 0.1 |
| G51D | 0.6 ± 0.3 | 0.8 ± 0.3 | 0.7 ± 0.3 | 1.1 ± 0.4 |
| A53E | 0.1 ± 0.1 | 0.2 ± 0.2 | 0.0 ± 0.0 | 0.2 ± 0.1 |
| A53T | 2.7 ± 1.3 | 19 ± 11 | 1.8 ± 0.7 | 15 ± 6.5 |
| A53V | 1.4 ± 0.3 | 6.8 ± 3.3 | 0.8 ± 0.2 | 8.1 ± 6.0 |
| V55Y | 2.8 ± 1.1 | 3.8 ± 2.9 | 2.1 ± 0.6 | 3.8 ± 2.0 |
| V66F | 0.5 ± 0.2 | 0.5 ± 0.4 | 0.3 ± 0.1 | 1.0 ± 0.4 |
| V74P | 0.6 ± 0.5 | 0.4 ± 0.3 | 0.2 ± 0.1 | 0.7 ± 0.5 |

*Data reported as mean cell infection ± standard deviation. i.c., intracranial; sc.n., sciatic nerve.*
