# Supplementary material for: A-synuclein prion strains differentially adapt after passage in mice
Source: PLoS Pathog. 2024 Dec 6;20(12):e1012746. doi: 10.1371/journal.ppat.1012746 (PMC11623799; doi:10.1371/journal.ppat.1012746)
Supplement: S4 Table — (DOCX) [file ppat.1012746.s009.docx]

**S4 Table. Infectivity of recombinant A53T preformed fibrils in cultured cells.**

| **Cell Line** | **Passaged control** | **A53T PFFs** |
| --- | --- | --- |
| A30G | 2.0 ± 0.8 | 11 ± 4.2 |
| E46K | 1.3 ± 0.4 | 4.9 ± 0.5 |
| K80E | 0.0 ± 0.0 | 2.7 ± 1.3 |
| G51D | 1.3 ± 0.7 | 19 ± 2.8 |
| A53E | 0.6 ± 0.2 | 12 ± 4.8 |
| A53T | 2.0 ± 0.4 | 47 ± 8.3 |
| A53V | 9.7 ± 2.0 | 77 ± 21 |
| V55Y | 2.8 ± 1.1 | 10 ± 5.6 |
| V66F | 0.6 ± 0.7 | 15 ± 3.9 |
| V74P | 0.3 ± 0.2 | 5.6 ± 3.0 |

*Data reported as mean cell infection ± standard deviation.PFFs, preformed fibrils.*
